# Supplementary figures and images for: Interspecific differences in the effects of masking and distraction on anti-predator behavior in suburban anthropogenic noise
Source: PLoS One. 2023 Aug 18;18(8):e0290330. doi: 10.1371/journal.pone.0290330 (PMC10437853; doi:10.1371/journal.pone.0290330)

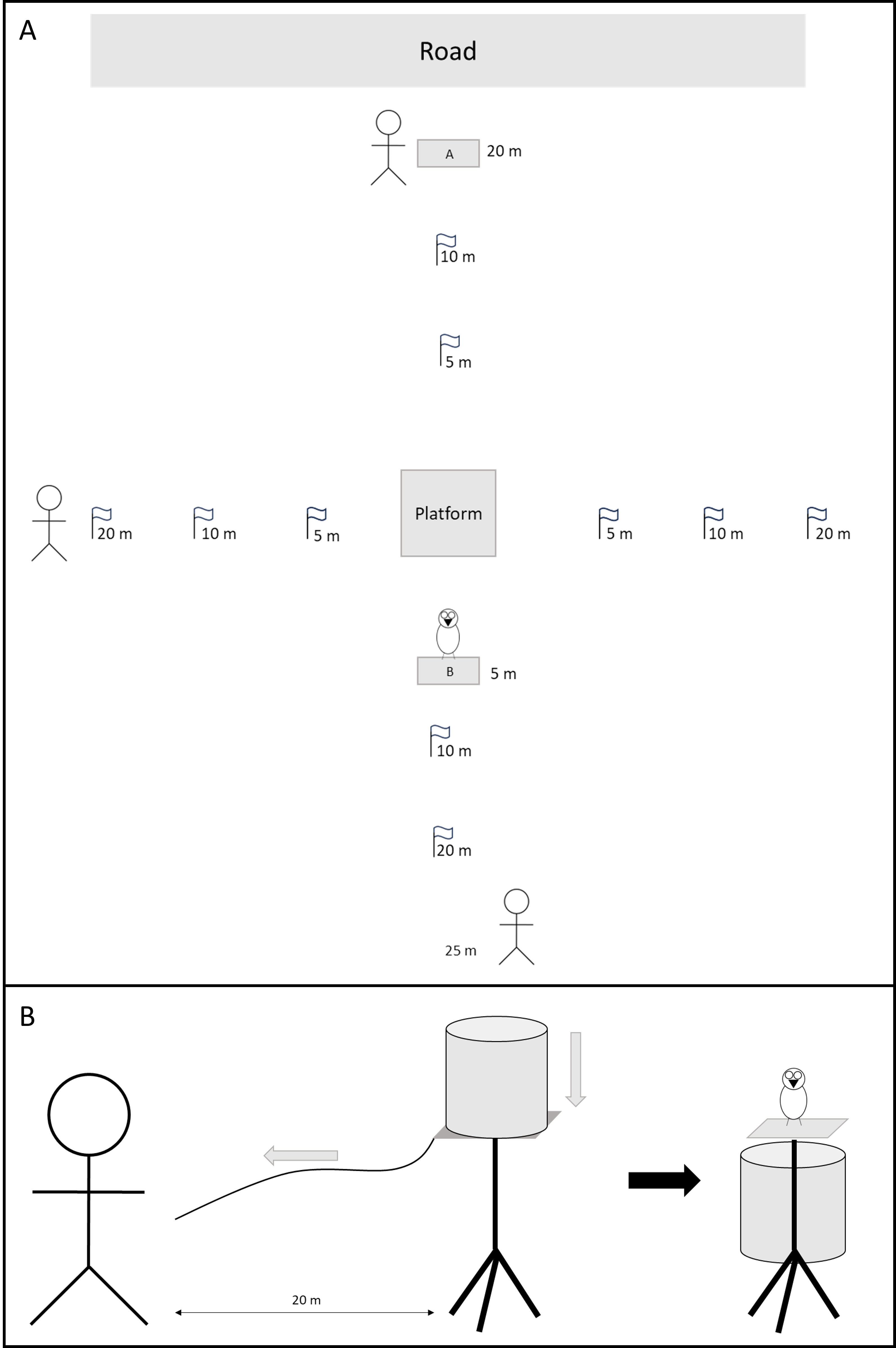

Supplement: S1 Fig — (A) Each trial location consisted of a feeding platform, an anthropogenic noise speaker 20 m from the platform in the direction of the nearest road, and a predator mount holding the owl or chickadee call speaker 5 m away from the platform in the opposite direction. Flags were placed around the platform at 5 m, 10 m, and 20 m away from the platform. One observer was located near the anthropogenic noise speaker, another behind the predator mount, and a third observer stood in the direction that gave them clearest view of the experimental area. All three observers were stationed outside the experimental radius. (B) The predator reveal mechanism was operated by an observer standing 20 m away, who pulled a string to remove supports beneath the camouflage cylinder, which would cause the cylinder to fall and reveal the predator stimulus. (TIF) [file pone.0290330.s001.tif]
